# When What's Left Is Right: Visuomotor Transformations in an Aged Population

**DOI:** 10.1371/journal.pone.0005484

**Published:** 2009-05-13

**Authors:** Lee A. Baugh, Jonathan J. Marotta

**Affiliations:** Perception and Action Lab, Department of Psychology, University of Manitoba, Manitoba, Canada; The University of Western Ontario, Canada

## Abstract

**Background:**

There has been little consensus as to whether age-related visuomotor adaptation effects are readily observable. Some studies have found slower adaptation, and/or reduced overall levels. In contrast, other methodologically similar studies have found no such evidence of aging effects on visuomotor adaptation. A crucial early step in successful adaptation is the ability to perform the necessary transformation to complete the task at hand. The present study describes the use of a viewing window paradigm to examine the effects of aging in a visuomotor transformation task.

**Methods:**

Two groups of participants, a young adult control group (age range 18–33 years old, mean age = 22) and an older adult group (age range 62–74, mean age = 68) completed a viewing window task that was controlled by the user via a computer touchscreen. Four visuomotor “flip” conditions were created by varying the relationship between the participant's movement, and the resultant on-screen movement of the viewing window: 1) No flip 2) X-Axis and Y-axis body movements resulted in the opposite direction of movement of the viewing window. In each of the 3) Flip-X and 4) Flip-Y conditions, the solitary X- or Y-axes were reversed. Response times and movement of the window were recorded.

**Conclusions:**

Older participants demonstrated impairments in performing a required visuomotor transformation, as evidenced by more complex scanning patterns and longer scanning times when compared to younger control participants. These results provide additional evidence that the mechanisms involved in visuomotor transformation are negatively affected by age.

## Introduction

Under normal circumstances, we have few problems using sensory information to respond to our environment. In fact, much of our ability to acquire and maintain the motor skills utilized on a daily basis capitalize on the ability to integrate and transform sensory information into a motor response. Previous research has shown that as one ages, there is a significant decline in the ability to acquire new motor skills, and a general decline in motor skill performance [1], [2], [3]. This finding is of special importance, as an aged population requires the learning of new motor skills, such as the use of a cane or wheelchair, to offset physical deterioration. While the cause of this diminished ability is most likely multi-faceted, a necessary requirement is the ability to successfully perform visuomotor transformations. Two primary mechanisms of adaptation have been (generally) agreed upon: *spatial realignment* and *strategic control* [4]. *Strategic control* includes modifying, selecting, or learning specific movement plans that are appropriate to the task at hand. Some relevant examples would be the use of side-pointing (a deliberate reach plan that misses in the manner opposite to the perturbation), on-line corrections to the movement path when feedback demonstrates the previously calculated movement plan is erroneous, and/or a temporary recalibration of local spatial arrangements for a specific task. The set of processes that are utilized during strategic control are most likely diverse, some of which are consciously accessible, and some of which are not. Relevant implicit knowledge of a novel visuomotor transformation has been construed as an internal model that approximates the transformation (the *internal model framework*). By capturing the new relationship between motor commands and their desired sensory outcomes, predictions related to the consequences of motor commands (forward model), and the motor commands required to achieve a desired output (inverse model) can be made [5], [6], [7], [8]. In contrast, *spatial realignment* is a result of the registered difference between the performance expected from a feed-forward movement plan and the performance achieved under feedback control, signalling a discrepancy between spatial maps. This discordance, in turn, provides the impetus for an incremental realignment that serves to improve performance [9].

Research has demonstrated declines in a wide range of cognitive abilities as a process of natural aging. For example, there are significant declines in verbal memory, spatial ability and verbal memory attributed to a reduction in processing speed [10]. It is likely that these declines in cognitive ability will have a substantial impact on visuomotor performance in a wide range of tasks. Previous studies examining the effect of aging on visuomotor transformation and adaptation have provided mixed results, with some studies demonstrating no age related impairments [11], [12], while other studies have shown slower and/or reduced adaptation [13], [14], [15], [16]. There are a number of possible explanations for this conflicting data, some of which may be inherent in the experimental task itself such as task transparency, participant instruction, and data analysis techniques, and others that are more a product of working with special populations, such as each participant's individual cognitive functioning.

The purpose of the present study is to further the research targeting the effects of aging and visuomotor transformation. Specifically, the current research is aimed at determining whether a healthy aged population performs at lower levels than younger controls in a visuomotor transformation task, a modified viewing window task [17]. The viewing window task is used as it has a number of benefits over previously reported visuomotor paradigms. Firstly, it is a substantially more complicated task than used previously, requiring an assortment of cognitive, visual, and visuomotor skills to complete. Second, it removes the focus of the experiment, at least in the mind of the participant, away from the visuomotor distortion, more naturally replicating the scenarios in which visuomotor transformation is required. This fact is perhaps a most notable distinction, as current research has previously demonstrated the effect explicit awareness of the perturbation can have on a participant's performance [18]. By increasing the task difficulty, requiring multiple neurological systems, and removing the focus of the task away from the visuomotor distortion per se, it may be possible to better understand the conflicting reports of aging effects on visuomotor transformation/adaptation.

Aged participants are expected to demonstrate marked difficulties in a number of dependent measures reflective of visuomotor transformation difficulty. When examining a participant's window movement scanpath, a more complicated pattern is expected when a visuomotor transformation is required, as evidenced by a larger number of reversals in each of the flip conditions. When examining average trial velocities, aged participants are expected to be slower overall when compared to young controls, and thus, age participants will take longer on a trial by trial basis. When examining the overall direction of movement of the window aged participants are hypothesized to initially deal with the required transformation through repeated direction reversals, leading to an increase in movement direction consistent with the direction of required transformation.

## Materials and Methods

### Ethics Statement

All experimental procedures received approval from the Psychology and Sociology Research Ethics Board of the University of Manitoba. All participants provided written informed consent before participating in any of the experiments.

### Subjects

The young control group consisted of twelve participants (2M, 10F; age range 18–33 years old, mean age = 22) recruited from the University of Manitoba's introduction to psychology participant pool and received course credit for their participation. The aged group consisted of eight participants (1M, 7F; age range 62–74, mean age = 68) recruited from the University of Manitoba Centre on Aging participant database and received no remuneration. Additionally, older adult participants were asked a series of questions to exclude those that had pre-existing conditions that may have affected performance (such as certain prescription medications and a history of stroke or arthritis, or the presence of neurological disorder), further, all aged participants were maintaining independent living status and reported their level of health to be excellent. Cognitive screening was performed on all participants. Six of the eight aged participants completed the Mini Mental State Exam [19] (mean score = 29.8, standard deviation = 0.41). Two participants were unavailable for an in-person screening, and instead completed an abbreviated mental test [20] (mean score = 10, standard deviation = 0). All cognitive screening scores were at or above normal levels [21]. All participants were right handed as assessed using a modified Edinburgh handedness inventory [22], and had normal or corrected-to-normal vision.

### Materials

#### Stimuli

Eighty common and easily identifiable objects were used for this experiment. Pilot work has established these items as being equally identifiable in both young and aged participants, with a mean correct identification of 74%, while using the viewing window paradigm. Additional pilot work examined the correlation between the time taken to identify objects in various visuomotor conditions with the associated KF-word frequency [23] values and found no significant relationship. Taken together, pilot data supports any differences in visuomotor distortion observed should not be confounded by item specifics.

Five of these objects were used for practice trials, with the remaining seventy-five being used for the experimental trials. Digital pictures of these objects were converted to greyscale format, and presented on a grey background. Outside of the viewing window, images were modified using a Gaussian blur algorithm to a level previously determined, ensuring all objects were equally difficult to identify and that no objects were recognizable based on peripheral information alone.

#### The Viewing Window

**Figure 1:**
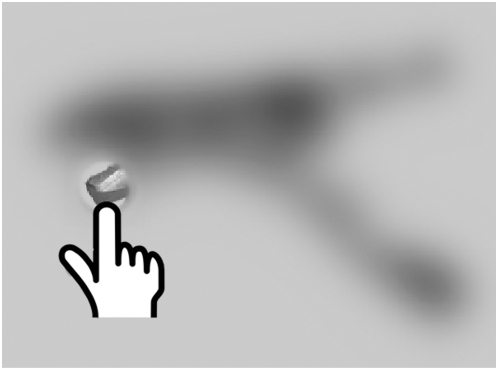
Viewing Window Illustration. The circular viewing window displays the underlying image in normal clarity, while the remainder of the image is heavily blurred.

The blurred images were displayed in the centre of the monitor. The “window” was a circular region with a 51 pixel length (1.3 cm) radius, covering a total of 8171 pixels. This area roughly corresponded with the size of useful foveal vision (2.98 degrees). The outermost region of the window displayed the underlying image at full blur. The innermost region displayed the image at normal clarity (See Figure 1), with a smooth transition between the two regions. This gradient border was used to provide a more natural viewing experience (for a complete description of the viewing window, see Baugh & Marotta, 2007). The viewing window was controlled by a touchscreen monitor, allowing the participants to move the window via a stylus held in their dominant hand, under their index finger. A stylus was used to ensure adequate pressure was maintained throughout the trial, and to eliminate smudges from the viewing area. The placement of the stylus directly under the tip of the index finger prevents the necessity to treat the stylus as a tool. The touchscreen allowed for a 1-1 correspondence between the participant's physiological movement, and the resultant on-screen movement of the focus-window over the presented object, allowing any visuomotor transformation requirement of the task to be under the control of experimental manipulation. In order to prevent the participant's hand from occluding the viewing window, the tip of the stylus corresponded with the bottom centre of the viewing area.

#### Procedure

Participants were tested individually at a station consisting of a personal computer, keyboard, and monitor. All stimuli were displayed on a 20.1” LCD monitor running at a resolution of 1600×1200 at 60 Hz. Overlaying the monitor was a Keytec “Magictouch” touchscreen. Participants were seated approximately 50 cm at eye-level from the monitor with both the keyboard and the touchscreen within easy reach.

Three Visuomotor-flip conditions were created by varying how the participant's body movements affected the onscreen movement of the viewing window. During the No Flip condition, the movement of the window was matched to the participant's hand movement. During the Full Flip condition, a participant's hand movement resulted in a reversed movement of the window. In each of the solitary X-Axis and Y-Axis Flip conditions, only one dimension (horizontal or vertical) of movement was reversed.

Participants were given both written and verbal instructions prior to beginning, and the correct use of the touchscreen and viewing-window were demonstrated by the experimenter. Participants were told that they could move a window around the screen, using a stylus held in their right hand, which would display the underlying object in perfect clarity. Participants were instructed to identify the presented object as quickly but as accurately as possible, and to signify their identification by pressing the space bar on the keyboard with their left thumb. Additionally, participants were told some of the trials would be difficult and that if they were unable to identify a given object, to take a “best-guess”. Following the participant's indication of object identification, the stimulus was removed from the screen and a message appeared prompting participants to type in their response using the keyboard, pressing the ‘Enter’ key when finished. Immediately following, the next trial would load. Participants were not given any feedback about their accuracy and continued through the entire experiment in a self-paced manner. Participants were naïve to the visuomotor manipulation. Object presentation and visuomotor-flip condition was randomized within subjects, with each visuomotor condition having a 25% chance of occurring on any given trial, eliminating any confounds between item and visuomotor condition. Items were presented only once. All participants began the experiment with five identical practice trials, and were limited to a 60 min testing session to reduce participant fatigue.

## Results

Due to aged participant's overall slower performance, four of the eight participants from this demographic were unable to complete the full set of 80 stimuli. For these participants, only data from the completed trials was included in later analyses. No significant differences in accuracy between the aged participants and the younger controls (average incorrect = 33.7%) were observed. Trials in which the target object was not correctly identified were analyzed separately from those in which correct identification was achieved.

Four separate examinations of the data from correct responses were conducted. First, participant's window movement scanpaths were examined. Next, an analysis on viewing window movement time was performed. Third, an analysis on average trial velocities was performed. Finally, an examination of the direction of movement was undertaken. An initial outlier analysis was performed on the movement time data, excluding values 2.5 standard deviations from each participant's mean for that condition. This resulted in the elimination of 2.8% of trials from all statistical analyses. Unless otherwise stated, multivariate analyses of variances were used, with an alpha level equal to .05.

Incorrect responses were submitted to the same four analyses previously described resulting in insignificant findings and are not reported.

### Sample Scanpaths

**Figure 2:**
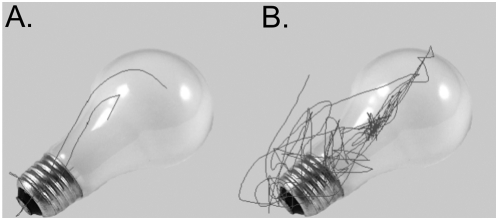
Example Scanpath. When no visuomotor transformation was required, aged participants (B) engaged in more scanning of the presented object than did young controls (A).

**Figure 3:**
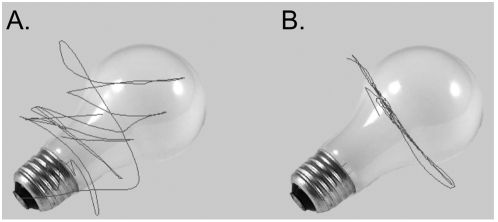
Example Scanpath – Flip in X-Axis. When a visuomotor transformation was required, aged participants (B) seemed to have greater difficulty in adapting to the new relationship of body movement and window movement (A).

**Figure 4:**
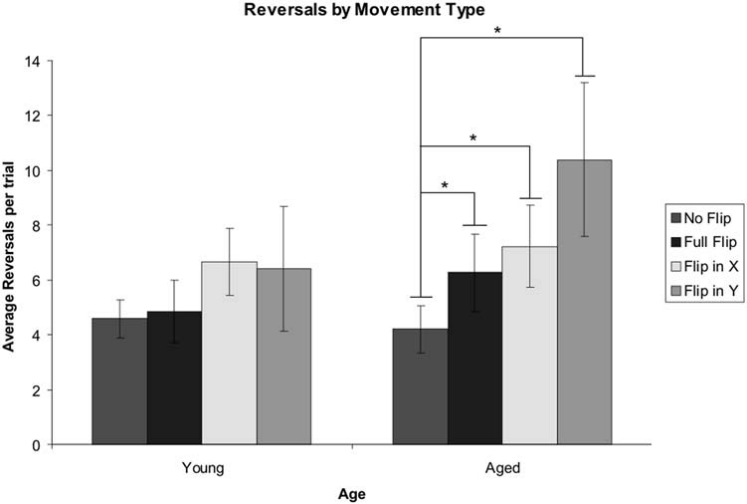
Number of Reversals. During an X- or Y-axis flip, the aged participants had significantly more reversals per trial than on trials when no visuomotor transformation was required. Error bars represent the standard error of the mean. *p<0.05 indicates a significant difference.

Upon visual inspection of a participant's individual scanpath, it was observed that when no visuomotor flip was required, aged participants engaged in more scanning with the window of the object than the young controls (Figure 2). Additionally, when a visuomotor-flip was required, aged participants appeared to have greater difficulty adapting to the new relationship between body and window movement (Figure 3). The number of reversals of window direction per trial was used as a measure of scanpath complexity, and can be seen in Figure 4. A reversal was considered to have occurred when an immediate change in both the X-Axis and Y-Axis direction of movement occurred. This dependent variable was selected as previous research has demonstrated this measure is consistent with the early phases of visuomotor adaptation [24].

A significant main effect of visuomotor condition was found, F(3,16) = 8.467, p = .001. Planned comparisons based on the expected effects of interest revealed significant differences between the normal condition and each of the solitary visuomotor flip conditions within the aged population (p's = .022 & .017). A flip along the x-axis was marginally different from the normal condition in the young participant group (p = .05).

### Total Movement Duration

**Figure 5:**
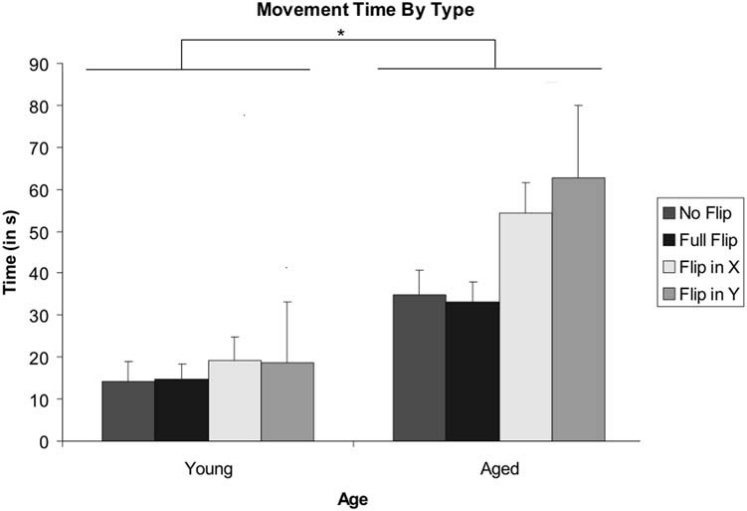
Average Movement Times. Overall, aged participants spent more time scanning the presented object when compared to the younger controls. Additionally, aged participants displayed a comparatively larger effect of both a flip in the X-Axis, and a flip in the Y-Axis, when compared to younger control participants, as evidenced by the significant Visuomotor Flip X Age interaction. Error bars represent the standard error of the mean. . *p<0.05 indicates a significant difference.

The participant movement time data (an aggregate value of the total amount of time the participant spent moving the viewing window, excluding time spent before window movement was initiatied) was separated into groups based on participant age (Young vs. Aged) and visuomotor-flip (Normal vs. Full Flip vs. Flip in X vs. Flip in Y). Data was compared using a multivariate ANOVA, treating participants as the random effect. A graphical representation of the data can be seen in Figure 5.

A main effect of visuomotor-flip was found, F(3,16) = 8.69, p<.001: Movement times were fastest in the normal condition, followed by the full flip, flip in X, and flip in Y, conditions respectively. Additionally, a main effect of age was found, with young controls taking less time scanning the objects than the aged participants (F(1,18) = 48.76, p<.000). Importantly, a significant interaction was observed between visuomotor-flip and age, characterized by the aged participants showing a proportionately larger effect of both a flip in X and a flip in Y, when compared to the younger control participants (F(3,16) = 3.5, p<.05).

### Movement Velocity

**Figure 6:**
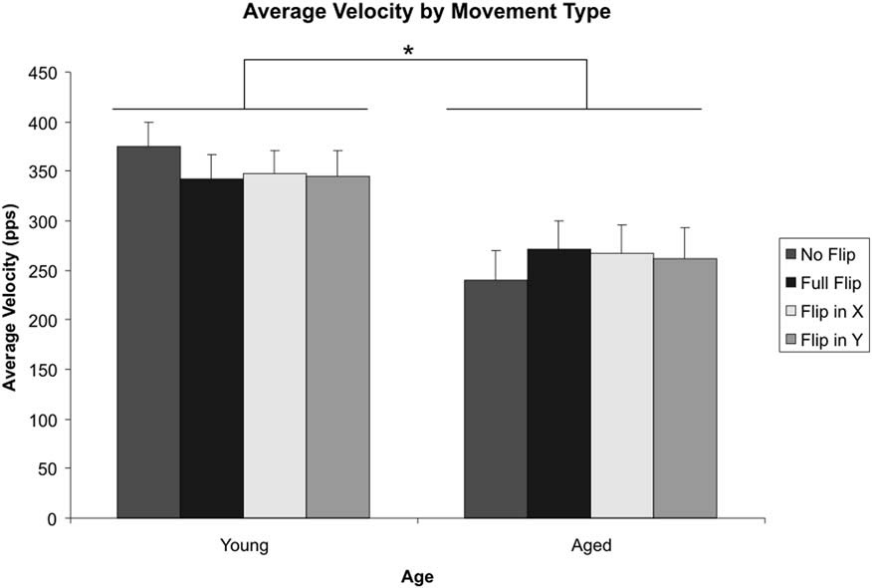
Average Trial Velocity. Aged participants had an overall lower mean velocity. However, there was no Age x Visuomotor interaction present. Error bars represent the standard error of the mean. *p<0.05 indicates a significant difference.

A second analysis of the data was conducted on movement velocity to ensure the effects observed were not accountable by aged participants moving the viewing window slower than younger controls. The average velocity of window movement (calculated in pixels per second) was calculated for the entire duration of each trial. A graph of the means for each of the four visuomotor conditions, by age, can be seen in Figure 6.

There was a significant between-subjects effect of age, consistent with the movement time analysis previously presented: Aged participants were moving significantly slower than the young controls (F(1,18) = 6.505, p<.05). There was no age by visuomotor flip interaction, and planned pairwise comparisons within the aged participants and young controls revealed no effects of visuomotor condition on movement velocity, providing evidence that the effects observed in the movement time data are not simply a result of the aged participants moving the window slower.

### Movement Direction

**Figure 7:**
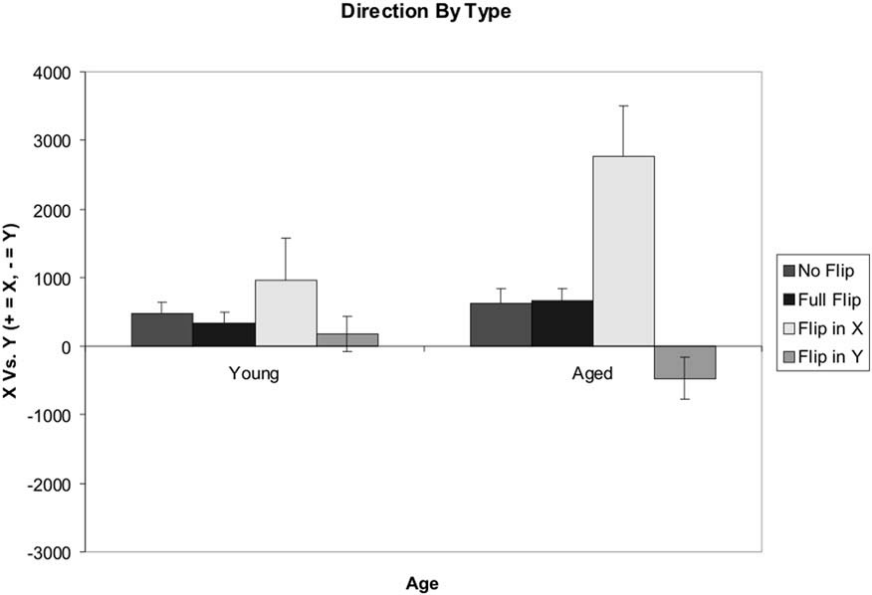
Direction of Movement (Summed X-Axis Movement – Summed Y-Axis Movement). Though not statistically significant, during an X-Axis or Y-Axis flip, the aged participants displayed more corresponding axis movement than did the younger controls. Error bars represent the standard error of the mean.

An analysis on the primary axis of movement by age in each of the four visuomotor flip conditions was conducted by taking the summed movement on the X-Axis and subtracting the summed movement from the Y-Axis. Therefore, positive values reflected a bias in moving the viewing-window along the X-Axis, whereas negative values reflected a bias towards moving the window along the Y-Axis. This data can be seen in Figure 7. Such an analysis was performed with the assumption that a visuomotor flip along a solitary axis would result in increased movement along that axis.

A main effect of visuomotor-flip was found (F(3,16) = 10.60, p<.000), as was a significant Age X Visuomotor-flip interaction (F(3,16) = 4.55, p<.05). Planned comparisons were conducted between the means of the young and aged participants in both an X-Axis flip and a Y-Axis flip, resulting in non-significant differences after correcting for multiple comparisons (using a Bonferroni correction).

## Discussion

The presented results demonstrate a number of interesting findings regarding the effects of natural aging on visuomotor transformation abilities. First, natural aging does affect viewing window performance when a visuomotor transformation is required to successfully complete the task. Visual examination of participant's scan path revealed a pattern and complexity not observed in the younger control participants. Specifically, aged participants had more complex scan paths (as reflected in the number of reversals). Data supports theories that posit the aged participants are having more difficulty in adapting to the new visuomotor map, as the number of sudden reversals and zigzags in movement path are reflective of ongoing / incomplete adaptation [24]. Statistical analyses revealed that aged participants took longer identifying objects by moving the window, as predicted. An analysis of movement velocity found a significant effect of age (with the older participants moving slower), but no age by visuomotor flip interaction. Thus, while aged participants were moving more slowly, this decrease in velocity does not fully account for the pattern of results observed in the movement times. When examining the overall direction of movement (as a preference towards horizontal or vertical scanning) an overall bias towards a left-right scanning pattern was observed, most likely a reflection of the privileged role of the horizontal axis in visual exploration [25], [26]. It could be this tendency towards left-right scanning that is responsible for an apparent axial asymmetry observed during the solitary flip conditions. In fact, the only time a left-right movement preference is substituted with a top-bottom axial preference is in the Y-Axis flip condition in the aged participants. Surprisingly, after adjusting for multiple comparisons, aged participants did not demonstrate increased movement along the direction of the visuomotor distortion. One plausible explanation is that the overall left-right scanning bias masked such effects from detection by increasing the variability of the data set.

Due to the self-paced methodology employed, there was the possibility of the two experimental groups to receive unequal exposure to the visuomotor distortion employed. However, it was only the young participant group that, on occasion, did not require the full hour to complete the task, and thus would receive a lesser amount of exposure. One would expect less exposure, or practice, with the visuomotor distortions used would lead to decreased performance rather than the converse, therefore any experimental confound would be weighted against the hypotheses presented.

The relative ease at which both younger and older participants were able to adapt to the “full-flip” condition warrants further discussion. Previous research examining screen-cursor rotations have found similar results: The cost associated with a 180 degree rotation (a full-flip) is significantly less than observed in other conditions, in a number of different measures, such as reaction time and error measurement [27], [28]. One explanation of this seemingly paradoxical finding relates to how feedback from visuomotor distortions may be generalized in terms of egocentric rotations [29], [30]. In the “full-flip” condition, an imagined 180 degree rotation of the self would result in performance equal to the “no-flip” condition. In comparison, both the “flip-x” and “flip-y” axis conditions, a single rotational strategy could not improve performance, and a more complicated process may be required. A second hypothesis explaining this effect concerns how the system recalibrates after a visuomotor discrepancy is detected. In the “full-flip” condition, all information relating hand movement to window movement is incorrect, and the system is best served by a full re-calibration. In both the “flip-x” and “flip-y” condition, only half of the information is flawed, making it necessary to selectively discard erroneous information, while retaining veridical relationships, which may be a more intensive process. Adaptation to visuospatial distortions appears to be implemented by learning rules that operate on separate parallel sensorimotor transformations. If enough of these rules are “broken” a gross recalibration of the visual-motor system must occur [31]. It seems, then, that the finding of no associated costs within the “full flip” condition fits well within the current body of research.

While interesting in isolation, the present study's results become more valuable when combined with contemporary literature involving parietal function in an aged population [32], [33], [34]. Ongoing research in our lab is currently verifying whether deficits in performance may be the result of decreased parietal functioning by utilizing an fMRI adapted paradigm similar to the one described here. It is important to note that strategic control processes are a form of adaptation often observed in the prism literature, and it is thought to play a pivotal role in initiating spatial realignment [4]. When one considers the nature of the viewing window task as it is presented here, satisfactory performance could be achieved by modifying, selecting, or learning specific movement plans that are appropriate to the given condition. For example, alternate movement plans could be learned and selected for each of the visuomotor distortion conditions confronted by the participant. It is therefore proposed that, in the aged population, decreased parietal functioning has reduced the participant's ability to adopt appropriate stratagem to deal with the visuomotor distortion.

Two apparent limitations are inherent in the presented research. First, the sample size of eight participants in the aged group is acknowledged as relatively small. While the sample size was chosen based on effect-size calculations obtained from pilot data, caution in generalizing the results is prudent and ongoing studies in our lab have increased this group size. Second, there was a pronounced asymmetry in sex representation within the two participant groups. Though in our small data set there were no apparent sex differences, previous research has suggested that male and female performance in spatial and visuomotor tasks may not be equal. For example, when performing complex visually guided movement sex differences have been demonstrated at both the behavioural and cortical level of analysis [35]. Further, this result may persevere into the later years of life [36], [37], at least in behavioural tasks.

This research provides support for previous experiments demonstrating similar deficits using an assortment of methodological procedures, and extends the current body of research to include a more cognitively demanding, yet less transparent visuomotor task. This extensionincreases the external validity of methods of testing visuomotor performance in an aging population, while conforming to important previously established findings. Buch et al. (2003) compared aged and younger control task performance using two types of visuomotor distortion tasks: One was an explicit, effortful, strategic task, consisting of a sudden rotation of the relationship between finger movement and resultant on-screen cursor movement. The second was an implicit, non-strategic task (consisting of a gradual rotation). In congruence with the results reported here, aged participants demonstrated a decline in task performance in the overt strategic task. Unfortunately, within the literature many studies were not specifically designed to evaluate functioning of either strategic control OR spatial adjustment, making dissociations between the two difficult. However, a deficit is often observed in aged participant's performance in tasks requiring a controlled, effortful application of specific movement in which the participant is overtly aware of the requirement [15], [16], [38]. The viewing window task is unique in a number of ways that may better serve to demonstrate the effect of aging on visuomotor control. First, the task is driven by an underlying goal that does not emphasize the required visuomotor transformation: In order for participants to correctly identify the presented object, they must move the window into an area of interest, but this transformation is not highlighted as the primary focus of the task. This situation intuitively seems more natural than a paradigm in which the visuomotor relationship is the prime focus of the participant, as we would seldom engage in these compensatory mechanisms if not for some purpose beyond the required transformation itself. Second, the viewing window task is both cognitive and motor in nature. A single viewing window trial requires selecting areas of the image that are of interest, adapting to a novel visuomotor relationship (if present), active exploration of the object, and ultimately decision processes to determine the object identity. The added complexity of task completion may place additional demands on the aging brain making any visuomotor deficiencies apparent. Previous research has presented similar findings, with deficits in similar tasks more apparent during dual-task scenarios as opposed to performing a sensorimotor transformation alone [39]. In ongoing research, our lab is examining individual components of the viewing window task in isolation of one another to pinpoint where deficits in performance may occur, however, as real world scenarios require the utilization of these multiple systems in conjunction on a daily basis, studying the sum process is a necessity.

The presented findings also have a number of clinical implications, in addition to supplementing our knowledge of how the visuomotor system ages. First, after a patient has suffered from a stroke, shaping procedures are often used to restore, or at least improve, visuomotor performance. While the present study does not directly rule out this line of therapy, it does suggest that the natural aging process may reduce the efficiency of such treatment regimes. Since many shaping procedures rely on explicit task instruction, and, assumingly, strategically controlled movement plan development, elderly patients may have an inherent deficit in performance. In fact, there have been some reports within the literature suggesting overt, explicit tasks may be less effective in fostering rehabilitation of motor skills than those that rely on implicit cues, lending credence to such hypotheses [40], [41]. Similarly, the results of the present study may offer insight into how best to target treatment, capitalizing on preserved abilities while avoiding or compensating deficient systems.

This research is of special importance to aging research. Due to the necessity for many elderly to use tools to assist in day-to-day living, a better understanding of visuomotor functioning in this population is required. The use of a wheelchair, cane, or any other such device inherently requires a spatial transformation, and in order to provide training and support to use these tools to their maximum advantage, the ability to incorporate these devices into visual and motor computations should be further assessed. The results presented here suggest an aged population may require special consideration when determining how effective or easy to utilize a particular assistance tool may be.

Finally, it is left to be seen if tasks such as the viewing window can fulfill a restorative role. Plasticity, in relevant terms for this discussion, can be thought of as a neuronal ability allowing sensorimotor systems to adapt to novel situations, and to maintain that adaptation for a period of time following. Research on aging has determined that plasticity remains throughout the lifespan in rat [42], [43] although there may be a decline in advanced age. Work specific to humans has, for the most part, supported animal models, with a surprising amount of neuronal plasticity remaining throughout the lifespan [44], [45], [46], [47]. It may, therefore, be possible, due to preserved plasticity, for extensive training to have a rehabilitative role. The present research demonstrated natural aging reduces visuomotor transformation performance in a relatively short (80 trial) span. It is currently an open question as to whether multiple and/or prolonged exposure to these visuomotor distortions increase task performance in the elderly, and if so, if practice is generalized beyond the specific task to real world scenarios.
